# Early and Definitive Diagnosis of Toxic Shock Syndrome by Detection of Marked Expansion of T-Cell-Receptor Vβ2-Positive T Cells

**DOI:** 10.3201/eid0903.020360

**Published:** 2003-03

**Authors:** Yoshio Matsuda, Hidehito Kato, Ritsuko Yamada, Hiroya Okano, Hiroaki Ohta, Ken’ichi Imanishi, Ken Kikuchi, Kyouichi Totsuka, Takehiko Uchiyama

**Affiliations:** * Tokyo Women’s Medical University School of Medicine, Tokyo, Japan

**Keywords:** toxic shock syndrome, T-cell-receptor, Vβ2-positive T cells, dispatch

## Abstract

We describe two cases of early toxic shock syndrome, caused by the superantigen produced from methicillin-resistant *Staphylococcus aureus* and diagnosed on the basis of an expansion of T-cell-receptor Vβ2-positive T cells. One case-patient showed atypical symptoms. Our results indicate that diagnostic systems incorporating laboratory techniques are essential for rapid, definitive diagnosis of toxic shock syndrome.

Toxic shock syndrome (TSS) is a severe illness caused by methicillin-resistant *Staphylococcus aureus* (MRSA) infection, usually during menstruation but also in the postpartum period. *S. aureus* produces several superantigenic exotoxins, including TSS toxin-1 (TSST-1), which activate a vast number of T cells in a T-cell-receptor Vβ-selective manner (*1*,*2*). Cytokines produced by T cells and activated by TSST-1 are thought to cause the abnormal changes of TSS (*1*,*2*). Polymerase chain reaction (PCR) analysis of peripheral blood T cells from adults with TSS has shown a protracted expansion of TSST-1–reactive Vβ2-positive T cells persisting for 4–5 weeks (*3*). TSS in neonates, referred to as neonatal TSS-like exanthematous disease, has been shown by flow cytometric analysis to involve an expansion of T-cell–receptor Vβ2-positive T cells (*4*,*5*).

Because many cases do not satisfy the strict diagnostic criteria for TSS proposed by the Centers for Disease Control and Prevention (*6*), revised clinical diagnostic criteria for TSS, including probable cases, have been proposed (Table) (*7*). In Japan, several clinicians have described a TSS-like clinical entity that could not be diagnosed as TSS even according to the revised criteria.

**Table:** Laboratory data on admission of case-patients

	Case-patient 1 Day 2 postpartum	Case-patient 2 Day 7 postpartum
**Laboratory findings (normal range)**		
Leukocytes (µL) (5,000–8,500)	2,800	17,500
Platelets (x104/µL) (13–40)	12.2	19.8
C-reactive protein (mg/dL) (0–0.4)	46.4	22
Total protein (g/dL) (6.5–8.2)	3.8	5.9
Albumin (g/dL (3.8–5.1)	1.6	3
Aspartate aminotransgerase (IU/L) (0–35)	30	51
Alanine aminotransgerase (IU/L) (0–35)	10	53
Lactic dehydrogenase (IU/L) (200–450)	712	698
Blood urea nitrogen (mg/dL) (5–12)	29.5	12.4
Creatine (mg/dL) (<0.8)	2.58	0.72
Uric acid (mg/dL) (1.2–4.5)	9.2	2.5
Natrium (mEq/L) (136–145)	135	133
Potassium (mEq/L) (3.5–5)	4.6	3.3
Chloride (mEq/L) (98–108)	104	99
Creatine kinase (IU/L) (10–70)	244	
Prothrombin time (sec) (12–14)	11.7	13.7
Activated partial thrombospilastin time (sec) (24–36)	36.7	43.2
Fibrinogen (mg/dL) (400–650)	674	668
Antithrombin-III (%) (70–120)	60	82
Fibrinogen degeneration product (µg/mL) (<10)	8.8	11.7
D-dimer (µg/mL) (<0.2)	5.22	3.93
Thrombin/antithrombin complex (ng/mL) (<3.0)	40	20.4
**Criteria for definite TSS (all criteria must be present)**	No	Yes
>38.9°C	Yes	Yes
Rash with desquamation		Yes
Hypotension <90mmHg	Yes	Yes
**Clinical or laboratory abnormalities (>3 organs)**		
Gastrointestinal		
Hepatic	Yes	Yes
Muscular		
Mucous membrane		Yes
Renal	Yes	Yes
Cardiovascular		
CNS		
**Criteria for probable TSS**	No	Yes
>3 criteria and desquamation or >5 criteria without desquamation		
>38.9°C	Yes	Yes
Rash		Yes
Hypotension	Yes	Yes
Myalgia		
Vomiting and/or diarrhea		
Mucous membrane inflammation		Yes
Clinical or laboratory abnormalities		
>2 organs		
Gastrointestinal		
Hepatic	Yes	Yes
Muscular		
Mucous membrane		Yes
Renal	Yes	Yes
Cardiovascular		
Central nervous system		

We report two cases of TSS with puerperal infection that could be diagnosed at the early stage of the clinical course by detecting a marked expansion of T-cell–receptor Vβ2-positive T cells, as measured by flow cytometric analysis. The symptoms of one patient were too complex to permit diagnosis according to the clinical criteria without evaluation of the TSST-1-reactive T cells. We discuss the role of T-cell analysis in peripheral blood mononuclear cells in the diagnosis of TSS.

## Case Reports

### Case 1

A 29-year-old Japanese woman underwent a cesarean section at a private clinic after premature membrane rupture. On postpartum day 3, shock with hypotension (67/37 mmHg) developed. No rash occurred during this period. She was transferred to the Maternal and Perinatal Center, Tokyo Women’s Medical University Hospital.

On admission, the patient was awake and alert, but her face was pale. Her body temperature was 37°C, blood pressure was 104/80 mmHg, heart rate was 140 bpm, and respiratory rate was 28 times/min. A pelvic examination showed a brownish discharge from the cervix. The uterus was approximately 10 x 10 cm in diameter, with no tenderness. Her systolic blood pressure subsequently decreased to 80 mmHg, respiratory rate increased to 44 times/min, and body temperature rose to 39°C. Mild hypoxemia (pO_2_ = 65 mmHg while breathing room air) became apparent, and the cardiothoracic rate shown on a chest x-ray film had increased to 54%. To treat shock, dopamine and fresh frozen plasma were administered with antithrombin III and antibiotic therapy (initially, pentocillin 2 *g*/day + panipenem/betamipron 1 *g*/day + amikacin 100 mg/day, and subsequently, panipenem/betamipron 1 *g*/day + vancomycin 1 *g*/day). The results of laboratory tests led to a suspected diagnosis of septic shock with disseminated intravascular coagulopathy (Table). Therefore, ulinastatin and gabexate mesilate, which possess both antifibrinolytic and anticoagulative effects, were administered (*8*,*9*). Because the patient’s general condition did not improve, she was admitted to the intensive care unit.

On day 3 after admission to the intensive care unit, an abscess was observed around the surgical wound. Bacteriologic tests showed that the vaginal discharge was positive for MRSA, and a preliminary diagnosis of TSS was made. Peripheral blood mononuclear cells were stained with antibodies to CD3, CD4, CD8, and T-cell-receptor–Vβ2 elements and examined for the percentage of Vβ2-positive T cells by a flow cytometer as described (*4*,*5*). Five hours after staining, a marked expansion of Vβ2-positive–T cells, unrelated to the CD4:CD8 ratio, was confirmed (Figure), indicating the definitive diagnosis of TSS in the early clinical course. Rash and desquamation, important clinical symptoms of TSS, did not develop. On day 7 after admission, the patient was discharged from the intensive care unit and entered the general ward. On day 17, intrapelvic abscess was incised and drained. Subsequently, her general condition improved. The percentage of Vβ2-positive–T cells decreased gradually over the course of 5 weeks (Figure). She has no long-term sequelae. MRSA isolated from this patient was later confirmed to be positive for TSST-1.

**Figure:**
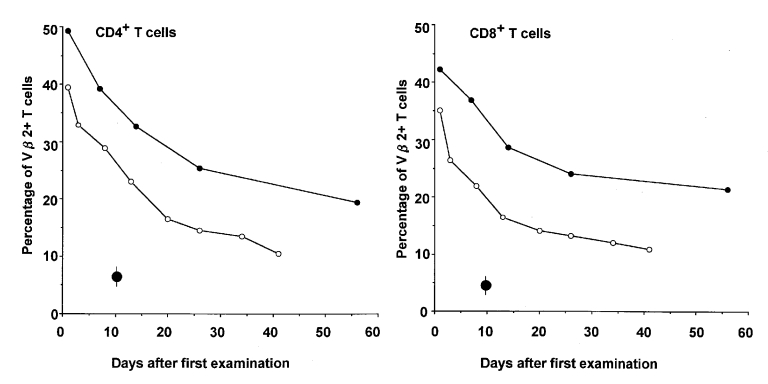
Results of T-cell–receptor Vβ2 positive T cells in two women with Toxic Shock Syndrome. The percentages of Vβ2^+^ CD4^+^ cells (left panel) and Vβ2 positive CD8 positive T cells (left panel) in peripheral blood mononuclear cells were determined after admission. Patient 1 (open circles) and patient 2 (open squares) refer to the same cases as in the text and Table ; ● represents mean±standard deviation in healthy adults (four men and three women).

### Case 2

A previously healthy 33-year-old Japanese woman had a fever (38.4°C) 1 week after an uncomplicated spontaneous vaginal delivery without episiotomy at a private clinic. She was transferred to Tokyo Women’s Medical University Hospital. On admission, the patient was awake and alert. Her body temperature was 38.8°C, blood pressure was 80/52 mmHg, heart rate was 127 bpm, and respiratory rate was 28 times/min.

A diffuse erythematous rash was present on the chest. It spread to the face and extremities and resolved after 8 days. A pelvic examination disclosed a brownish discharge from the cervix. The uterus was approximately 6 cm x 8 cm in diameter, with no tenderness. The laboratory test results and clinical symptoms suggested a diagnosis of TSS (Table). To treat the hypotension, dopamine and fresh frozen plasma were administered. The patient also received antithrombin III, ulinastatin, and gabexate mesilate as well as antibiotics (initially, imipenem/cilastatin 2 *g*/day + amikacin 200 mg/day, and subsequently, vancomycin 2 *g*/day).

One day after admission, bacteriologic tests showed that the vaginal discharge and breast milk were positive for MRSA. On day 5 after admission, peripheral blood mononuclear cells were examined for expansion of Vβ2 T cells. A marked expansion of Vβ2 T cells was confirmed (Figure), indicating the definitive diagnosis of TSS. The Vβ2-positive T cells gradually diminished to normal levels. Desquamation of the extremities occurred on day 11 after admission. From day 3 after admission, the patient’s general condition improved gradually, and she was discharged on day 14. She had no long-term sequelae. Isolates of MRSA isolated were later found to be TSST-1–positive.

## Discussion

Puerperal infection is a major cause of maternal death. Postpartum nonmenstrual TSS has received attention as a potential cause of puerperal infection (*10*–*12*). Knowing the incidence of MRSA infections in medical institutions would be helpful. For example, Fujino et al. reported that 246 MRSA isolates were obtained from 74 inpatients in December 2000 in a Tokyo hospital with 27 wards and 925 beds (*13*). During the past 4 years, no TSS cases have occurred in our department of obstetrics and gynecology. The two patients in this report were transferred from a private clinic. Although the incidence of TSS is rare in our department, we are concerned that the incidence is not rare in small private clinics. Our two cases may provide important clues to the actual incidence of TSS in women with puerperal infection.

In both of our patients, the diagnosis of TSS was confirmed during acute illness on the basis of expansion of TSS-1-reactive Vβ2 T cells in peripheral blood mononuclear cells. Diagnosis solely on the basis of clinical symptoms was not possible in case 1 because of the absence of skin rash and desquamation, cardinal symptoms of TSS, and the presence of signs of severe multiple organ failure. In case 2, diagnosis of TSS was straightforward because of typical clinical symptoms. The severe multiple organ failure in case 1 may have suppressed the development of skin reactions. Our report strongly suggests that some TSS cases that cannot be correctly diagnosed because of a complicated clinical picture. Our results indicate that diagnostic systems incorporating laboratory techniques are essential for the rapid, definitive diagnosis of TSS.

Our experience suggests the necessity for better estimates of the incidence of postpartum staphylococcal infections, TSS associated with MRSA, and TSS that does not satisfy generally accepted diagnostic criteria. Several clinical trials in fields other than obstetrical infections with MRSA are now underway in Japan to address these issues.
